# Characteristics of Electric Scooter and Bicycle Injuries After Introduction of Electric Scooter Rentals in Oslo, Norway

**DOI:** 10.1001/jamanetworkopen.2022.26701

**Published:** 2022-08-15

**Authors:** August Vincent Stray, Henrik Siverts, Knut Melhuus, Martine Enger, Pål Galteland, Ingar Næss, Eirik Helseth, Jon Ramm-Pettersen

**Affiliations:** 1 Department of Maxillofacial Surgery, Oslo University Hospital, Norway; 2 Division of Orthopaedic Surgery, Oslo University Hospital, Norway; 3 Institute of Clinical Medicine, Faculty of Medicine, Oslo University Hospital, Norway; 4 Department of Neurosurgery, Oslo University Hospital, Norway

## Abstract

**Importance:**

When society introduces and accepts new transportation modes, it is important to map risks and benefits.

**Objective:**

To compare electric scooter (e-scooter) and bicycle injuries.

**Design, Setting, and Participants:**

This cohort study is based on prospectively collected data on Norwegian patients who sustained e-scooter or bicycle injuries and presented to an emergency department affiliated with Oslo University Hospital between January 1, 2019, and March 31, 2020.

**Main Outcomes and Measures:**

e-Scooter and bicycle injuries were evaluated for associations with sex, age, time of injury, helmet use, intoxication, body region, and injury severity. Descriptive statistics are presented as mean (SD) or number with percentage, with significance set at *P* < .05 (2-tailed).

**Results:**

During the study period, 3191 patients were included (850 e-scooter riders, 2341 bicyclists) with 3839 injuries recorded (997 e-scooter, 2842 bicycle). The mean (SD) age of those injured was 34 (17) years, 2026 riders (63.5%) were male, 1474 (46.2%) were helmeted at the time of injury, and 516 (16.2%) were intoxicated by alcohol or other drugs. The annual incidence of injuries was 120 per 100 000 inhabitants for e-scooters and 340 per 100 000 inhabitants for bicycles. Men were overrepresented in both groups (529 e-scooter riders [62.2%] and 1497 bicyclists [63.9%]). e-Scooter riders were younger than bicyclists (mean [SD] age, 31 [12] vs 35 [18] years). Most injured e-scooter riders were aged 20 to 40 years, whereas injured bicyclists had a broader age distribution. e-Scooter injuries commonly occurred on weekends (378 [46.6%]) and during evening (230 [32.3%]) or nighttime (242 [34.1%]) hours. Most bicycle injuries occurred during weekdays (1586 [69.7%]) and daytime (1762 [61.3%]). e-Scooter riders were more often intoxicated (336 [39.5%] vs 180 [7.7%]) and had a lower rate of helmet use (18 [2.1%] vs 1456 [62.2%]). During nighttime, 230 injured e-scooter riders (91.3%) and 86 bicyclists (69.4%) were intoxicated. e-Scooter riders had more head and neck (317 [31.7%] vs 636 [22.4%]) and lower-limb (285 [28.6%] vs 632 [22.2%]) injuries and fewer upper-limb (341 [34.2%] vs 1276 [44.9%]), thoracic (41 [4.1%] vs 195 [6.9%]), and abdominal, pelvic, and lumbar (13 [1.3%] vs 103 [3.6%]) injuries.

**Conclusions and Relevance:**

In this cohort study, e-scooter riders were younger than bicyclists, did not use helmets, were more often intoxicated, and were more often injured during nighttime. The rate of intoxication among e-scooter riders injured at night was high. Preventive measures, including awareness campaigns, regulating e-scooter availability, improving infrastructure, and implementing stricter helmet and alcohol policies, may prove effective for reducing injuries.

## Key Points

**Question:**

How do electric scooter (e-scooter) injuries compare with traditional bicycle injuries?

**Findings:**

In this cohort study of 3191 patients with e-scooter or bicycle injuries, e-scooter injuries commonly occurred at nighttime and involved young adults who were not helmeted and most often intoxicated. In contrast, most bicycle injuries were sustained during commuting hours and involved riders of a wider age range who were often helmeted and less likely to be riding while intoxicated.

**Meaning:**

The rate of intoxication in e-scooter riders injured at nighttime is high, and introduction of preventive measures should be considered.

## Teaser

This cohort study uses emergency department data to compare characteristics of injured electric scooter (e-scooter) and bicycle riders after the introduction of e-scooter rentals in Oslo, Norway.

## Introduction

Electric scooter (e-scooter) rentals were introduced in Oslo, Norway, in March 2019 and have become a popular mode of intracity transportation according to the Norwegian Institute of Transport Economics.^1^ Shortly after the introduction of e-scooter rentals, it became clear that this new transportation mode came with some downsides, such as a high risk for injuries when used by persons riding while intoxicated and low rates of helmet use among e-scooter riders in general.^2,3,4,5,6,7,8,9,10,11^ These concerns are also relevant for bicyclists. However, despite the injury risk associated with cycling, bicycle riding overall is positively associated with public health.^12,13,14,15^ Although it seems unlikely that e-scooters offer any cardiovascular benefits that directly improve public health, they may indirectly contribute positively to public health by being a climate-friendly alternative to cars. However, in 2020, the Norwegian Institute of Transport Economics reported that e-scooters replace walking in 60% of cases, bicycles in 6%, public transportation in 23%, and cars in only 8%. The report concluded that e-scooters at present do not offer any obvious climate benefits, but that this may change in the future.^1^

When society introduces a new transportation mode and accepts it as part of daily life, it is important to know its risks and benefits. In this study, we compare characteristics (ie, age, sex, helmet use, alcohol or drug intoxication, time and type of injury) associated with injuries from riding e-scooters vs bicycles in patients presenting at a 24/7 downtown emergency department affiliated with Oslo University Hospital (OUH). The information presented may form the basis for injury prevention measures.

## Methods

In this cohort study, we prospectively collected data on behalf of the Norwegian Public Road Administration and Directorate of Health. Some of the data included have been used by the Norwegian Public Roads Administration for a government report on injury prevention.^16^ All patient information was collected with verbal consent. In accordance with Norwegian legislation, the Office of the Privacy and Data Protection Officer of OUH approved the study (case 19/05317) as an audit project with anonymous data, which is exempt from the obligation to report to the Data Inspectorate and Regional Committee for Medical and Health Research Ethics. The study was performed in accordance with the Declaration of Helsinki. This study follows the Strengthening the Reporting of Observational Studies in Epidemiology (STROBE) reporting guideline.

Oslo is a modest-sized European capital with approximately 697 000 inhabitants. Oslo University Hospital is the major trauma care center in the city. The Department of Orthopedic Emergency (DOE) is a satellite branch of OUH and functions as a 24/7 walk-in outpatient clinic that treats minor to moderate trauma and is the only public outpatient emergency clinic in Oslo. Patients with severe injuries are transported directly to OUH.^17^

The study compares patients who sustained e-scooter and bicycle injuries and presented to the DOE during a 1-year period. The DOE prospectively registered all e-scooter injuries from April 1, 2019, to March 31, 2020, and all bicycle injuries from January 1, 2019, to December 31, 2019. The dates of data collection differ because of the introduction of e-scooter rentals in March 2019. Patients admitted to the DOE after e-scooter and bicycle accidents were asked whether they wore a helmet and whether they consumed alcohol or other drugs before riding (herein referred to as intoxication). We also reviewed and registered injuries that occurred within the boundaries of the Oslo municipality and for which patients were brought directly to the OUH trauma center. A registrar entered this information along with a description of the accident circumstances and injuries into a local database.

The main outcome of this study was to compare characteristics of patients with e-scooter vs bicycle injuries. The 2 transportation modes have similarities in area of application and are therefore considered relevant and important for comparison. We extracted the following information from the database: sex, age, time of injury (daytime, 6:00 am to 4:59 pm; evening, 5:00 pm to 10:59 pm; nighttime, 11:00 pm to 5:59 am), 2-wheeled e-scooter or bicycle injuries (electric bicycles excluded), helmet use, intoxication, body region injured, and injury severity. If there was any uncertainty when obtaining a certain set of data (eg, time of injury), that specific category would be recorded as “system missing.”

Assessment of intoxication was based on patient information. A breathalyzer test was not administered, and blood samples were not routinely evaluated. The 5 body regions described in the database were the head and neck; thoracic; abdominal, pelvic, and lumbar; upper-limb; and lower-limb regions. Severity was categorized according to standardized guidelines (Felles minimum data set [FMDS] index^18^) defined by the Norwegian Ministry of Health. The FMDS index is hinged on the Abbreviated Injury Scale (AIS); therefore, anatomic injury was coded according to this scale. Abbreviated Injury Scale severity scores range from 1 (minor) to 5 (critical) to 6 (maximum [eg, decapitation]). Total injury severity is calculated as the Injury Severity Score (ISS), which is the sum of the squares of the single highest AIS severity score in each of the 3 most severely injured ISS body regions. The ISS ranges from 1 to 75, with serious injury classified as an ISS of 9 or higher and severe injury as an ISS of 16 or higher. In the FMDS index, minor injury corresponds to an AIS score of 1, moderate to a score of 2, and severe to scores 3 to 6.^18^

### Statistical Analysis

Statistical analyses were performed using SPSS, version 25 (IBM Corporation) and Excel for Mac, version 16.49 (Microsoft Corporation). Descriptive statistics are presented as mean (SD) or number with percentage. A 2-tailed *P* < .05 by χ^2^ test was considered significant.

## Results

During the study period, 3191 patients were included (850 e-scooter riders and 2341 bicycle riders) with a total of 3839 injuries recorded (997 e-scooter and 2842 bicycle). The mean (SD) age was 34 (17) years; 2026 patients (63.5%) were male, 1165 (36.5%) were female, 1474 (46.2%) were wearing a helmet, and 516 (16.2%) were intoxicated at the time of injury (Table 1). The main body regions injured were the upper-limb (1617 [42.1%]), head and neck (953 [24.8%]), lower-limb (917 [23.9%]), thoracic (236 [6.1%]), and abdominal, pelvic, and lumbar (116 [3.0%]) regions. The number of patients with 2 or more injuries was 877 (27.5%) (226 [26.6%] e-scooter riders and 651 [27.8%] bicyclists).

**Table 1.** Patient Characteristics of Injured Electric Scooter (e-Scooter) Riders and Bicyclists

Characteristic	No. (%)			*P* value^a^
	All (N = 3191)	e-Scooter (n = 850)	Bicycle (n = 2341)	
Mean (SD) age, y	34 (17)	31 (12)	35 (18)	<.001
Sex				
Female	1165 (36.5)	321 (37.7)	844 (36.1)	.38
Male	2026 (63.5)	529 (62.2)	1497 (63.9)	
Intoxication				
Yes	516 (16.2)	336 (39.5)	180 (7.7)	<.001
No	2582 (80.9)	484 (56.9)	2098 (89.6)	
Unknown	93 (2.9)	30 (3.5)	63 (2.7)	
Helmet				
Yes	1474 (46.2)	18 (2.1)	1456 (62.2)	<.001
No	1485 (46.5)	707 (83.8)	778 (32.2)	
Unknown	232 (7.3)	125 (14.7)	107 (4.6)	
No. of injuries^b^	3839	997	2842	
Injury severity^c^				
Minor	2554 (66.5)	675 (67.7)	1879 (66.1)	.06
Moderate	988 (25.7)	233 (23.4)	755 (26.6)	
Severe	296 (7.7)	89 (8.9)	207 (7.3)	
Body region				
Head and neck	953 (24.8)	317 (31.7)	636 (22.4)	<.001
Thorax	236 (6.1)	41 (4.1)	195 (6.9)	<.001
Abdomen, pelvis, lumbar spine	116 (3.0)	13 (1.3)	103 (3.6)	<.001
Upper limb	1617 (42.1)	341 (34.2)	1276 (44.9)	<.001
Lower limb	917 (23.9)	285 (28.6)	632 (22.2)	<.001

^a^ *P* values highlight any significant differences between the e-scooter and bicycle groups.

^b^ 651 bicyclists and 226 e-scooter riders had 2 or more injuries.

^c^ Injury severity was categorized on the basis of the Felles minimum data set, a standardized index defined by the Norwegian Ministry of Health.^18^ The index is hinged on the Abbreviated Injury Scale (AIS). In this study, minor injury corresponds to AIS score 1, moderate injury to AIS score 2, and severe injury to AIS scores 3 to 6.

No significant differences in severe injuries were found between e-scooter riders and bicyclists (89 [8.9%] vs 207 [7.3%], respectively; *P* = .09). Two or more severe injuries were sustained by 28 (3.3%) e-scooter riders and 51 (2.2%) bicyclists.

**Figure 1.**
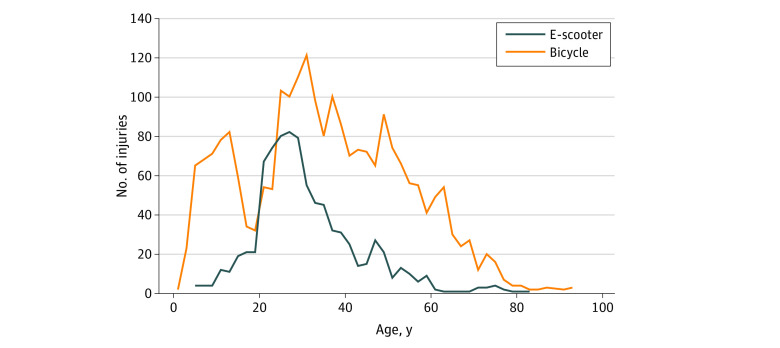
Age Distribution Between Electric Scooter (e-Scooter) and Bicycle Groups Most injured e-scooter riders were aged 20 to 40 years, whereas most injured bicyclists were aged 25 to 60 years. e-Scooter injuries were rare in children. A shift toward increasing prevalence of e-scooter injuries is seen in the late teens, whereas bicycle injuries show a marked dip.

**Figure 2.**
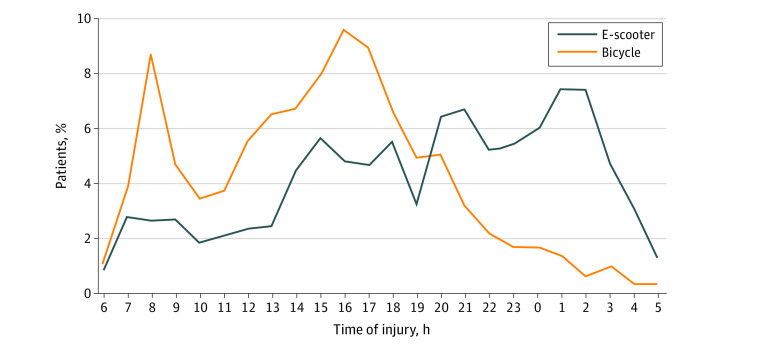
Timeline of Electric Scooter (e-Scooter) and Bicycle Injuries During 24 Hours Most bicycle injuries occur during rush hour, whereas most e-scooter injuries occur during evening and nighttime. Time is based on a 24-hour clock from 6:00 AM to 5:00 AM.

Annual incidence of injuries was 120 per 100 000 inhabitants for e-scooters and 340 per 100 000 inhabitants for bicycles. No significant sex differences were found between e-scooter riders and bicyclists (529 [62.2%] vs 1497 [63.9%]; *P* = .38). e-Scooter riders were younger than bicyclists (mean [SD] age, 31 [12] vs 35 [18] years). Most injured e-scooter riders were in the age range of 20 to 40 years (Figure 1). The bicycle group had a broader age distribution, which peaked at age 25 to 55 years and showed a marked dip at approximately 20 years of age (Figure 1). Adolescents younger than 18 were involved in 71 (8.4%) e-scooter accidents and 483 (20.6%) bicycle accidents, whereas children younger than 12 were involved in 19 (2.4%) e-scooter accidents and 307 (13.1%) bicycle accidents.

e-Scooter injuries often occurred on weekends (378 [46.6%]), whereas most bicycle injuries occurred during weekdays (1586 [69.7%]). The majority of e-scooter injuries occurred during the evening and nighttime (between 5:00 pm and 10:59 pm, 230 [32.3%]; between 11:00 pm and 5:59 am, 242 [34.1%]), whereas most bicycle injuries occurred during the daytime (between 6:00 am and 4:59 pm, 1762 [61.3%]). Daytime bicycle injuries peaked during the morning and afternoon rush hours (Figure 2).

e-Scooter riders, compared with bicyclists, were more often intoxicated (321 [39.5%] vs 180 [7.7%]) and had a lower rate of helmet use (18 [2.1%] vs 1456 [62.2%]) (Table 1). Among patients aged 16 years or older (thereby adjusting for the higher proportion of children in the bicycle group), 333 (41.5%) were intoxicated in the e-scooter group, and 180 (9.4%) were intoxicated in the bicycle group. During nighttime hours, 230 (91.3%) injured e-scooter riders and 86 (69.4%) bicyclists were intoxicated at the time of injury (Table 2). e-Scooter riders compared with bicyclists had more head and neck injuries (317 [31.7%] vs 636 [22.4%]), more lower-limb injuries (285 [28.6%] vs 632 [22.2%]), fewer upper-limb injuries (341 [34.2%] vs 1276 [44.9%]), fewer thoracic injuries (41 [4.1%] vs 195 [6.9%]), and fewer abdominal, pelvic, and lumbar injuries (13 [1.3%] vs 103 [3.6%]) (Table 1).

**Table 2.** Rate of Intoxicated Electric Scooter (e-Scooter) Riders and Bicyclists According to Time of Day and Weekday vs Weekend

	No. of intoxicated/total No. of riders (%)^a^	
	Weekday	Weekend
Total		
e-Scooter	119/434 (27.4)	219/378 (57.9)
Bicycle	68/1586 (4.2)	111/691 (16.1)
Daytime		
e-Scooter	19/257 (7.4)	8/92 (8.7)
Bicycle	26/1309 (2.0)	16/453 (3.5)
Evening		
e-Scooter	32/99 (32.3)	49/112 (43.8)
Bicycle	23/237 (9.7)	28/154 (18.2)
Nighttime		
e-Scooter	68/78 (87.2)	162/174 (93.1)
Bicycle	19/40 (47.5)	67/84 (79.8)

^a^ Of 812 e-scooter riders studied, 38 had missing data, and of 2277 bicyclists studied, 64 had missing data.

## Discussion

In this cohort study, injured e-scooter riders, compared with injured bicyclists, were younger, were more often intoxicated, had a lower rate of helmet use, and were more often injured during nighttime. Male riders were overrepresented for both e-scooter and bicycle injuries, which is consistent with previous reports.^19,20,21,22^ The main reason for male overrepresentation is most likely a gender difference in everyday risk-taking behavior.^23^

Injured e-scooter riders were younger than bicyclists. Most e-scooter injuries involved patients aged 20 to 40 years, whereas bicycle injuries tended to occur in all age groups. Children and adolescents younger than 18 years were involved in 8.4% of e-scooter accidents and in 20.6% of bicycle accidents. A possible explanation for this difference in age distribution is that most Norwegian e-scooter companies enforce age restrictions that only permit rentals to people who are older than 16 or 18 years. Renting an e-scooter also requires mobile payment, which may be a limitation for children. In California, which also has age restrictions on e-scooter rentals, Trivedi et al^2^ investigated e-scooter injuries and reported that patients younger than 18 years were involved in 11% of e-scooter accidents, which is consistent with our findings. Trivedi et al also indicated that self-enforced regulations imposed by private e-scooter companies may be inadequate. In part, we agree with this sentiment because there is always room for improvement; however, when comparing e-scooter accidents with bicycle accidents, it seems that current age regulations could in fact contribute to measurable and positive effects with respect to reducing the number of children and adolescents involved in e-scooter accidents. The low number of senior citizens involved in e-scooter accidents compared with bicycle accidents could be related to an inverse relationship between age and willingness to explore novel technology, better understanding of the risks associated with e-scooter riding, and lower risk-taking behavior.

Only 2.1% of the injured e-scooter riders in our study were wearing a helmet, which is consistent with previous reports.^3,5,7,8,9,10,11^ The observed differences between e-scooter riders and bicyclists with respect to helmet use and intoxication are most likely due to several factors, such as the instant availability of rental e-scooters in Oslo. As a passive form of transportation, e-scooters require minimal physical effort and no need to change clothing or freshen up after a ride, making them a convenient alternative for unplanned travel. Unplanned travel is more likely to occur outside working hours, such as traveling to or from a social happening. Riding while intoxicated and low adherence to helmet use could also be associated with instant availability. Bicycles, on the other hand, are less available as rentals, and riding them usually requires a more conscious action. We believe this makes bicycles more suitable for planned travel, such as commuting to and from work. This assumption correlates well with the observed peak of bicycle injuries during commuting hours.

Head and neck injuries were more frequent among e-scooter riders, and upper-limb injuries were more frequent in bicyclists. Both findings agree with results from other studies.^5,24,25,26,27^ The high number of head and neck injuries among e-scooter riders compared with bicyclists is concerning. Our study gives no clear explanation for this difference. However, it is tempting to attribute this difference at least partly to the very low rate of helmet use among e-scooter riders. For bicyclists, helmet use has an established role in preventing traumatic brain injury,^28,29,30,31,32^ and riding while intoxicated is significantly associated with less helmet use.^33,34^ Thus, the influence of alcohol or drugs could indirectly contribute to an increased number of head injuries due to the association between intoxication and helmet use. Other studies that have described e-scooter–related head injuries underline the association between these injuries and intoxication and the lack of helmet use.^5,6,35^

This study and other recent studies from Finland and Australia indicate a high number of e-scooter injuries.^3,5^ Potential injury prevention measures are awareness campaigns (especially targeting teens), improvement of road infrastructure (separate lanes for e-scooters and bicycles), standardized lighting on e-scooters, mandatory helmet use, similar alcohol limits as applied for other motorized vehicles, restricting nighttime use of e-scooters, and time- or area-specific speed regulations. We also believe that advocating for the continuation and, if possible, improvement of current enforcement of the age restriction on rental e-scooter use could be beneficial toward preventing injuries among children and adolescents.

### Strengths and Limitations

The main strength of this study was the prospective collection of data. The external validity of this study is to some extent restricted to cities comparable with Oslo. When comparing this study with others, one must be aware that bicycling and e-scooter riding are associated with local conditions, such as topography, climate, and policy regulations.

A limitation is reliance on self-reported intoxication, although response rates indicate a large degree of honesty (answers were anonymous and not eligible for legal prosecution). The phrasing in the registration form used to collect data on intoxication does not discriminate between alcohol and other drugs. Therefore, although alcohol was largely the reported substance patients admitted being under the influence of, this study does not differentiate between alcohol and other drugs. Another potential limitation is that e-scooter rentals were introduced in Oslo in March 2019; thus, the registration period of e-scooter injuries in this study was set to April 1, 2019, to March 31, 2020. Bicycle injuries were registered from January 1, 2019, to December 31, 2019. Considering the likelihood of a novel transportation mode needing time to gain general popularity, it is plausible that the numbers of e-scooter injuries in April and perhaps also May are lower than to be expected. Otherwise, we do not believe that the 3-month lag in registration period had a considerable impact because both groups were recorded over a 1-year period.

## Conclusions

In this cohort study of individuals who were injured while using e-scooters or bicycles in Oslo, e-scooter riders, compared with bicyclists, were younger, were less frequently wearing a helmet, were more often intoxicated, and were more often injured at night. The rate of intoxication in nighttime injured e-scooter riders was high. Given these findings, a preventive benefit may be gained by introducing measures such as improving infrastructure, initiating awareness campaigns targeting teens, regulating e-scooter numbers and availability at night, implementing helmet regulations, and enforcing stricter alcohol policies. Continued age restriction is also likely to keep the number of children involved in accidents low.
